# A "Memorial" Hospital

**Published:** 1919-11-15

**Authors:** 


### A "Memorial" Hospital.

THE Leicester Royal Infirmary, whose progressive building policy has been noted frequently in THE HOSPITAL, is to have the orthopædic and isolation hospital, the need for which was referred to at the quarterly meeting of the Governors, reported on page 85 of our issue of October 25. The Ministry of Pensions asked for the establishment of the former building more than a year ago, but the proposal never eventuated, and the need for the latter building was accentuated by the recent influenza epidemic, which was very acute in Leicester.

The source of the new provision is contained in a letter from the solicitors to the estate of the late Mr. Thomas George Langham, who, with his family, have been long associated with agricultural interests in the county. The executors are desirous of associating the name of their testator in a permanent manner with a suitable memorial, and they propose to allot, from funds in their hands, a sum of £20,000 to the infirmary, which they desire should be applied in the provision of an orthopædic and isolation hospital, to be erected in the grounds of the institution, the testator's name to be associated therewith. The proposal has been most gratefully accepted by the Board of Governors at a special meeting, and steps will be taken immediately to proceed with the building, which will contain about fifty beds—one floor being used for each of the two departments.

The provision of this new hospital definitely raises the question of the enlargement of the Nurses' Home by some fifty beds. This matter also came under consideration at the meeting of the board, and the progressive policy of the institution received confirmation in the decision to proceed with this important project also. The outlay involved in the erection of these new buildings will be approximately £40,000; and, in view of the need of enlarged accommodation, and of the opinion which the board hold that building costs are not likely to be lower for at least two years, it is proposed to proceed with the two schemes side by side, and immediately.

It is interesting to record that the policy of the board received endorsement from the City Council, when Alderman J. Chaplin, the newly elected Mayor, referred to the good work of the charitable institutions of the city, and particularly to the Leicester Royal Infirmary. The new buildings would bring the infirmary right into the forefront of modern hospitals.

